# Intraoperative circulation predict prolonged length of stay after head and neck free flap reconstruction: a retrospective study based on machine learning

**DOI:** 10.3389/fonc.2024.1473447

**Published:** 2025-01-10

**Authors:** Zhongqi Liu, Jinbei Wen, Yingzhen Chen, Bin Zhou, Minghui Cao, Mingyan Guo

**Affiliations:** ^1^ Department of Anesthesiology, Shenshan Medical Central, Sun Yat-sen Memorial Hospital, Sun Yat-sen University, Shanwei, China; ^2^ Department of Anesthesiology, Sun Yat-sen Memorial Hospital, Sun Yat-sen University, Guangzhou, China; ^3^ Department of Oral and Maxillofacial Surgery, Sun Yat-sen Memorial Hospital, Sun Yat-sen University, Guangzhou, China

**Keywords:** intraoperative circulation, time series data, machine learning, free flap reconstruction, prolonged length of stay

## Abstract

**Background:**

Head and neck free flap reconstruction presents challenges in managing intraoperative circulation, potentially leading to prolonged length of stay (PLOS). Limited research exists on the associations between intraoperative circulation and PLOS given the difficulty of manual quantification of intraoperative circulation time-series data. Therefore, this study aimed to quantify intraoperative circulation data and investigate its association with PLOS after free flap reconstruction utilizing machine learning algorithms.

**Methods:**

804 patients who underwent head and neck free flap reconstruction between September 2019 and February 2021 were included. Machine learning tools (Fourier transform, et al.) were utilized to extract features to quantify intraoperative circulation data. To compare the accuracy of quantified intraoperative circulation and manual intraoperative circulation assessments in the PLOS prediction, predictive models based on these 2 assessment methods were developed and validated.

**Results:**

Intraoperative circulation was quantified and a total of 114 features were extracted from intraoperative circulation data. Quantified intraoperative circulation models with a real-time predictive manner were constructed. A higher area under the receiver operating characteristic curve (AUROC) was observed in quantified intraoperative circulation data models (0.801 [95% CI, 0.733–0.869]) compared to manual intraoperative circulation assessment models (0.719 [95% CI, 0.641–0.797]) in PLOS prediction.

**Conclusion:**

Machine learning algorithms facilitated quantification of intraoperative circulation data. The developed real-time quantified intraoperative circulation prediction models based on this quantification offer a potential strategy to optimize intraoperative circulation management and mitigate PLOS following head and neck free flap reconstruction.

## 1 Introduction

In the realm of microsurgery, free flap transplantation has emerged as a standard technique for reconstructing head and neck defects resulting from maxillofacial tumors resection and osteonecrosis. This procedure presents certain characteristics that pose challenges in managing intraoperative circulation and can potentially hinder postoperative rehabilitation. These include prolonged duration, extensive wounds, and intricate and delicate procedures (1).

The duration of hospitalization after surgery, referred to as length of stay (LOS), serves as a crucial metric evaluating the quality encompassing free flap reconstruction and postoperative rehabilitation. Prolonged length of stay (PLOS) strongly links to increased healthcare expenses and elevated postoperative complications, impacting patients’ quality of life (2). However, patients’ average LOS might surpass 10 days (3). Hence, optimizing rehabilitation methods and reducing LOS following free flap reconstruction becomes crucial. The influence of perioperative events on patients’ LOS is apparent in addition to surgical procedures (4), and efforts made in perioperative management have a crucial role to play in preventing PLOS (5–7).

Intraoperative circulation management, as an important part of perioperative management of free flap reconstruction, clearly deserves attention. Previous works have reported that in non-cardiac surgery, aberrant intraoperative circulatory state, including intraoperative hypotension (8–11), rapid heart rate (12, 13), abnormally elevated blood pressure (14) affect patients’ prognosis (myocardial injury, renal damage, 30-day mortality, etc.) and increase incidence of PLOS (15). To better appraise intraoperative circulation, variability of blood pressure, time-weighted hypotension (16, 17) and hypertension (17) were developed in addition to the above. However. the influence of intraoperative circulation during free flap reconstruction on the occurrence of PLOS has been rarely documented, warranting further exploration.

In addition, intraoperative circulation is made up of time series data. The traditional assessment variables mentioned earlier have limitations in effectively reflecting the variability of intraoperative circulation during surgery and the complex interconnections between these variables. Xue et al. reported that the association between intraoperative circulation and postoperative adverse events in non-cardiac surgery can be evaluated more accurately using the time-series data assessment metrics (18). Furthermore, the emergence of machine learning algorithms in the medical field has provided novel solutions for evaluating and modifying the intraoperative circulation data. Hatib F et al. developed a machine-learning-based predictive model for early warning of intraoperative hypotension based on arterial pressure waveforms (19), which was replicated and found to be effective by Wijnberge. M. et al. (20). Furthermore, using machine learning algorithms like Fourier transformation and Ricker wavelet analysis, extracting features for time series data processing was no longer a challenging task (21–23).

Therefore, employing machine learning algorithms, the purpose of this retrospective study was to assess and evaluate the predictive impact of quantified intraoperative circulation data on head and neck surgery with free flap reconstruction. It was hypothesized that the use of machine learning algorithms would provide a more thorough view of the relationship between intraoperative circulation data and PLOS after free flap reconstruction. The first aim of this study was to extract intraoperative circulation data eigenvalues using machine learning algorithms for their quantification. The second aim of this study was to investigate the potential superiority of quantified intraoperative circulation data over manual assessment metrics in predicting PLOS.

## 2 Materials and methods

### 2.1 Ethics

Ethical approval was obtained from the Institutional Review Board (IRB) of Sun Yat-sen Memorial Hospital, Sun Yat-sen University.

### 2.2 Study design

The medical records of patients who underwent head and neck surgery with free flap reconstruction at Sun Yat-sen Memorial Hospital between September 2019 and February 2021 were collected and randomly assigned into primary and validation cohorts in a ratio of 8:2 in the present retrospective study. Eligibility criteria required individuals to have received head and neck surgery with free flap reconstruction during the designated period. And exclusion criteria were as follows: missing values of demographic characteristics, perioperative laboratory examination data, surgical or fluid variables, missing intraoperative blood pressure, heart rate or pulse values for > 10 min, and patients younger than 9 years old.

Before anesthesia induction, arterial blood pressure was measured invasively with an arterial catheter placed into the radial artery and was recorded together with heart rate and pulse at 5-minute intervals. Arterial blood pressures, heart rates, and pulse values were linearly interpolated between readings (11). In the present study, mean arterial pressure (MAP) was calculated from systolic and diastolic blood pressure. The threshold of hypertension and hypotension was defined as 30% above and below the baseline MAP (MAP before induction), respectively. Time-weighted (TW) hypertension during surgery was calculated as the product of the depth of hypertension above the threshold of hypertension (mmHg) multiplied by the time above the threshold of hypertension (min). Similarly, TW hypotension during surgery was calculated as the depth of hypotension below the threshold of hypotension (mmHg) × the time below the threshold of hypotension (min). Intraoperative average real variability (ARV) and squared version of the generalized ARV (ARVs) of MAP were calculated by the following formula (16):


$$\text{ARV}=\frac{1}{T}\sum_{k=1}^{N-1}t\left|MAP_{k+1}-MAP_{k}\right|$$

$$\text{ARVs}=\frac{1}{T}\sum_{k=1}^{N-1}\frac{{\left|MAP_{k+1}-MAP_{k}\right|}^{2}}{t_{k+1}-t_{k}}$$

T was the total time between the first and last MAP reading, N is the number of MAP readings and t is the time interval between each set of readings, MAP_k_ and MAP_k+1_.

Meanwhile, to quantify intraoperative circulation time series data, machine learning-based technologies (including Fourier transform, Ricker wavelet, Lempel-Ziv compression, approximation entropy, permutation entropy, linear regression following blocks aggregation, and percentage of duplicate and non-duplicate values) were implemented with a dual test fade discover rate (FDR) of 0.01 in the current work to obtain the eigenvalues from patients’ intraoperative circulation time series data.

In the current study, each patient received a standardized anesthetic approach that included sevoflurane and opioids (sufentanil and remifentanil) for maintenance, as well as vasopressors if the patient experienced prolonged hypotension.

### 2.3 Data collection

From patients’ medical records, demographic information such as sex, age, body mass index (BMI), the reason for the flap (benign or malignant tumor, osteoradionecrosis), flap types (fibular flap, anterolateral thigh flap, posterior tibial artery flap, radial forearm flap, or others), American Society of Anesthesiologists (ASA) status, smoking history, radiotherapy history, and comorbidities (hypertension, diabetes, stroke, coronary heart disease, and others) were collected. The BMI was caculated using the height and weight of the patients.

Preoperative lab examination data, including hemoglobin (Hb), albumin, serum C-reactive protein (CRP), and differential blood cell counts, were collected seven days before surgery. Data from postoperative laboratory examinations, including Hb, albumin, and differential blood cell counts, were gathered within one day after surgery. Based on the blood cell counts, the perioperative neutrophil-to-lymphocyte ratio (NLR) and platelet-to-lymphocyte ratio (PLR) were computed.

Surgical variables included intraoperative blood loss, duration of surgery, vasopressor administration, intraoperative blood transfusion, urine output and postoperative ICU admission. The conscious decision to use vasopressors (i.e. norepinephrine, dopamine or ephedrine) on a case-by-case basis was made by the anesthesia crew. A blood transfusion was required when the hemoglobin (Hb) level was lower than 70 g/L or the hematocrit (Hct) was lower than 25% in patients with uncompromised function (cardiac or pulmonary). A blood transfusion was indicated when Hct was less than 25% for patients younger than 60 years and less than 30% for patients older than 60 years in hemodynamically impaired patients.

Fluid variables included the volume and rate of both intraoperative infusion and 24-hour infusion (crystalloid, colloid and total). The infusion rate was standardized to the patient’s body weight (mL/[kg×hr]). Intraoperative fluid infusions were administered at the discretion of the anesthesiologists based on intra-arterial blood pressure monitoring (avoided the occurrence of intraoperative hypertension or hypotension), stroke volume variation (maintained between 10-15) and the patient’s urine output (maintained no lower than 1 mL/[kg×hr]). The surgical crew was responsible for titrating the rate of postoperative fluid infusions considering the patient’s heart rate, blood pressure, and urine output.

### 2.4 Outcome

Length of stay (LOS) stands for the total number of days between surgery and discharge, and PLOS stands for any length of stay above the median of LOS.

### 2.5 Statistical analysis

Continuous variables were presented as mean (SD) or median (quartiles) based on their normalcy. To summarize categorical variables, frequencies (percentages, %) were employed. Continuous variables were analyzed using the t-test or the Mann-Whitney U test, depending on their normality. Categorical variables were analyzed using the chi-square or Fisher exact test, depending on their frequency of occurrence.

Between the primary and validation cohorts, the univariable association of baseline demographic data, perioperative laboratory examination parameters, surgical factors, fluid variables and LOS was examined. The median LOS was used to divide patients into PLOS and Non-PLOS groups. The primary cohort’s PLOS and Non-PLOS groups were then compared using univariate and multivariate logistic regression analysis to determine risk factors for PLOS. Collinearity diagnostics were performed to determine the features for multivariable comparison.

Features derived from two intraoperative circulation data evaluation methods were estimated in the present study. One was the features of manual intraoperative circulation data evaluations which included the intraoperative TW hypertension, TW hypotension, ARV and ARVs. Another was the features extracted from intraoperative circulation time series data through machine learning-based tools (including Fourier transform, Ricker wavelet, etc.). Occasional missing points in intraoperative circulation data were filled by the average of adjacent data points. The min-max normalization was utilized for data pre-processing after intraoperative circulation data features extraction. To determine and compare the predictive value of these two intraoperative circulation data evaluation methods, random forest and xgboost algorithms were utilized incorporating features from different intraoperative circulation evaluation methods and other clinical features in the primary cohort to evaluate feature relevance and develop binary classification predictive models for PLOS after free flap reconstruction. The Shapley additive explanations (SHAP) algorithm was applied to our prediction models to evaluate the importance of features. The receiver operating characteristic curve (ROC) and confusion matrix was drawn, and the area under the receiver operating characteristic curve (AUROC) and overall accuracy (OA) was calculated to evaluate the accuracy of these predictive models. The eigenvalues of intraoperative circulation data extracted through machine learning algorithms dynamically evolved as the intraoperative circulation data accumulated. Correspondingly, the risk probabilities derived from predictive models incorporating these features varied accordingly. Therefore, these risk probabilities were referred to as prediction scores, which were systematically calculated and analyzed in this study.

Univariable and multivariable analysis was performed with IBM SPSS software (version 25.0; SPSS Inc, Chicago, IL). Machine learning algorithms (Fourier transform, ricker wavelet, Lempel-Ziv compression algorithm, random forest, xgboost, etc.) were performed with Python (version 3.8.5). Differences with a p< 0.05 significance level were deemed statistically significant.

## 3 Results

### 3.1 Patients and clinical characteristics

**Figure 1:**
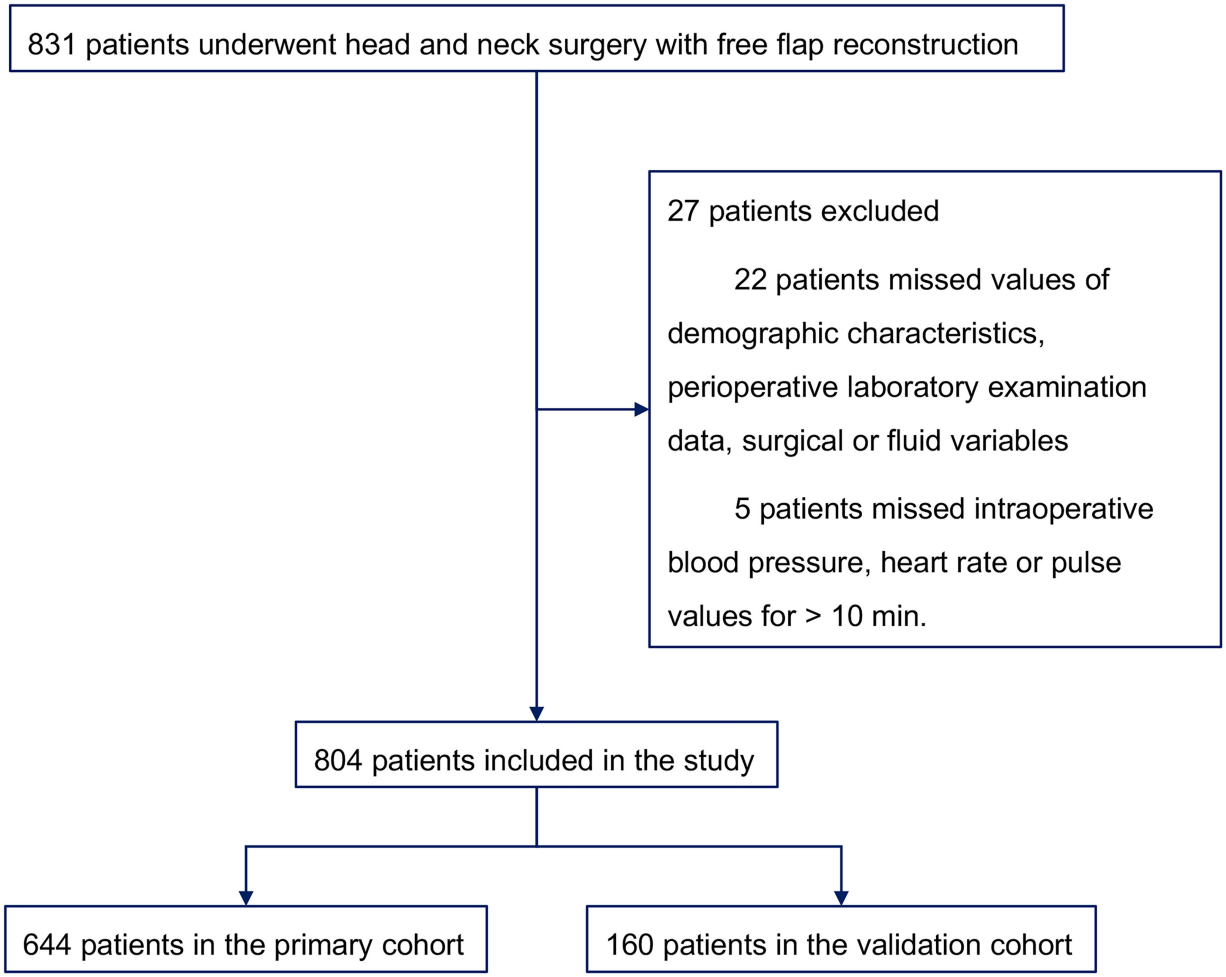
Study profile.

A total of 831 individuals who underwent head and neck surgery with free flap reconstruction were initially included. 22 patients were excluded for missing values on demographic characteristics, perioperative laboratory data, surgical or fluid variables, 5 patients were removed due to missing intraoperative blood pressure, heart rate or pulse values for > 10 min, resulting in a final enrollment of 804. Among them, 644 participants formed the primary cohort and 160 the validation cohort (**Figure 1**). Median LOS was 10 days, with 25% and 75% quartiles of 8 and 12 days, respectively. Those with LOS exceeding 10 days were classified as PLOS, while LOS no higher than 10 days were Non-PLOS cases. **Table 1** summarizes of the demographic characteristics of patients along with perioperative laboratory examination data, surgical and fluid factors for both the primary and validation cohorts.

**Table 1:** Univariate Analysis between the Primary and Validation cohorts.

	Primary Cohort (n=644)	Validation Cohort (n=160)	Univariate (*P* value)
Sex (male), No. (%)	419 (65.1)	98 (61.3)	0.368
Age, mean (SD), yr	55.83 (13.92)	54.60 (15.20)	0.327
Reason for Flap, No. (%)			0.277
Tumor	593 (92.1)	151 (94.4)	
Osteoradionecrosis	50 (7.8)	8 (5.0)	
Others	1 (0.2)	1 (0.6)	
BMI, mean (SD), kg/m^2^	22.50 (3.61)	22.31 (3.41)	0.549
Flap Types, No. (%)			0.418
Fibular Flap	199 (30.9)	42 (26.3)	
Anterolateral Thigh Flap	306 (47.5)	74 (46.3)	
Posterior Tibial Artery Flap	95 (14.8)	33 (20.6)	
Radial Forearm Flap	23 (3.6)	5 (3.1)	
Other	21 (3.3)	6 (3.8)	
ASA Status, No. (%)			0.219
I or II	342 (53.1)	86 (53.8)	
III	290 (45.0)	74 (46.3)	
IV	12 (1.9)	0 (0.0)	
Smoking Status, No. (%)	205 (31.8)	44 (27.5)	0.289
Comorbidities, No. (%)			
Hypertension	144 (22.4)	26 (16.3)	0.090
Diabetes Mellitus	60 (9.3)	12 (7.5)	0.471
Stroke	16 (2.5)	5 (3.1)	0.587
Coronary Heart Disease	18 (2.8)	6 (3.8)	0.525
Other	22 (3.4)	7 (4.4)	0.560
Total	195 (30.3)	41 (25.6)	0.247
Radiotherapy History, No. (%)	93 (14.4)	17 (10.6)	0.209
Preoperative			
Hemoglobin, mean (SD), g/L	132.39 (18.22)	133.06 (17.95)	0.678
Albumin, mean (SD), g/L	37.72 (4.70)	37.81 (4.46)	0.812
NLR, median (quartiles)	2.34 (1.67, 3.28)	2.47 (1.59, 3.49)	#0.620
PLR, median (quartiles)	146.01 (114.02, 198.53)	147.21 (116.54, 198.13)	#0.889
Postoperative			
Hemoglobin, median (quartiles), g/L	111.00 (98.00, 123.00)	110.00 (101.00, 122.00)	#0.699
Albumin, median (quartiles), g/L	30.40 (27.20, 33.30)	30.50 (27.60, 34.50)	#0.214
NLR, median (quartiles)	15.33 (10.01, 22.70)	16.28 (11.22, 26.44)	#0.103
PLR, median (quartiles)	251.64 (173.24, 366.69)	289.56 (192.53, 418.47)	#0.072
Blood Loss, median (quartiles), ml	300.00 (200.00, 400.00)	300.00 (200.00, 400.00)	#0.406
Duration of Surgery, median (quartiles), min	375.00 (290.00, 455.00)	365.00 (290.00, 465.00)	#0.670
Intraoperative Fluid Infusion, median (quartiles), ml	3000 (2500, 3500)	3000 (2500, 3500)	#0.953
Intraoperative Fluid Infusion Rate, median (quartiles), ml/(kg×h)	8.13 (6.47, 9.74)	8.42 (6.67, 10.25)	#0.104
Intraoperative RBC Transfusion, median (quartiles), u	0.0 (0.0, 2.0)	0.0 (0.0, 2.0)	#0.359
Intraoperative FFP Transfusion, median (quartiles), ml	0.0 (0.0, 200.0)	0.0 (0.0, 100.0)	#0.237
Intraoperative Urine Output, median (quartiles), ml	700.0 (450.0, 1000.0)	775.0 (500.0, 1000.0)	#0.229
Fluid Infusion over 24 hr, median (quartiles), ml	4200 (3500, 4900)	4300 (3650, 5000)	#0.431
Fluid Infusion Rate over 24 hr, median (quartiles), ml/(kg×h)	2.91 (2.39, 3.56)	3.08 (2.50, 3.70)	#0.119
Postoperative ICU Admission, No. (%)	42 (6.5)	10 (6.3)	0.900
Reoperation, No. (%)	39 (6.1)	7 (4.4)	0.413
Vasopressor Administration, No. (%)	146 (22.7)	31 (19.4)	0.368

BMI, Body Mass Index; ASA, American Society of Anesthesiologists; RBC, red blood cell; FFP, free-frozen plasma; NLR, neutrophil-to-lymphocyte ratio; PLR, platelet-to-lymphocyte ratio; LMR, lymphocyte- to-monocyte ratio.

The *P* value is derived from the univariable association analyses between the primary and validation cohorts.

# indicates that the Mann-Whitney U test was utilized.

### 3.2 Univariate and multivariate comparisons between the PLOS and Non-PLOS groups in the primary cohort

Within the primary cohort, uncovering differences between the PLOS and Non-PLOS groups, variables including Age, smoking status, history of hypertension, total comorbidities, preoperative albumin and NLR levels, postoperative hemoglobin and albumin levels, blood loss, duration of surgery, intraoperative RBC and FFP transfusion, fluid infusion rate over 24 hr, postoperative ICU admission, and reoperation emerged as significantly distinct following univariate comparisons (**Table 2**). Furthermore, smoking status (odds ratio [OR] = 0.566; 95% CI, 0.373–0.861; p = 0.008), intraoperative RBC transfusion (OR = 1.141; 95% CI, 1.005–1.295; p = 0.042) and postoperative reoperation (OR = 0.110; 95% CI, 0.038–0.323; p =<0.001) were identified as independent risk factors for PLOS in patients who underwent head and neck surgery with free flap reconstruction (**Table 2**).

**Table 2:** Univariate and Multivariate Comparisons between the PLOS and Non-PLOS Groups in the Primary Cohort.

	PLOS (n=340)	Non-PLOS (n=304)	Univariate (*P* value)	Multivariate [*P* value (OR; 95% CI)]
Sex (male), No. (%)	230 (67.6)	189 (62.2)	0.146	0;950 (1.013; 0.667 to 1.539)
Age, mean (SD), yr	56.93 (14.47)	54.60 (13.19)	0.034	0.656 (1.003; 0.988 to 1.019)
Reason for Flap, No. (%)			0.563	
Tumor	313 (92.1)	280 (92.1)		
Osteoradionecrosis	27 (7.9)	23 (7.6)		
Others	0 (0.0)	1 (0.3)		
BMI, mean (SD), kg/m^2^	22.28 (3.70)	22.74 (3.49)	0.106	0.962 (0.998; 0.937 to 1.064)
Flap Types, No. (%)			0.052	0.377
Fibular Flap	102 (30.0)	97 (31.9)		0.939 (1.039; 0.388 to 2.784)
Anterolateral Thigh Flap	175 (51.5)	131 (43.1)		0.477 (1.424; 0.538 to 3.772)
Posterior Tibial Artery Flap	38 (11.2)	57 (18.8)		0.994 (0.996; 0.345 to 2.874)
Radial Forearm Flap	14 (4.1)	9 (3.0)		0.341 (1.877; 0.514 to 6.856)
Other	11 (3.2)	10 (3.3)		
ASA Status, No. (%)			0.414	0.696
I or II	174 (51.2)	168 (55.3)		0.440 (1.769; 0.415 to 7.530)
III	158 (46.5)	132 (43.4)		0.517 (1.589; 0.391 to 6.464)
IV	8 (2.4)	4 (1.3)		
Smoking Status, No. (%)	127 (37.4)	78 (25.7)	0.001	0.008 (0.566; 0.373 to 0.861)
Comorbidities, No. (%)				
Hypertension	89 (26.2)	55 (18.1)	0.014	0.269 (0.671; 0.331 to 1.362)
Diabetes Mellitus	38 (11.2)	22 (7.2)	0.086	
Stroke	12 (3.5)	4 (1.3)	0.080	
Coronary Heart Disease	12 (3.5)	6 (2.0)	0.232	
Other	11 (3.2)	11 (3.6)	0.789	
Total	117 (34.4)	78 (25.7)	0.016	0.847 (0.937; 0.486 to 1.809)
Radiotherapy History, No. (%)	53 (15.6)	40 (13.2)	0.381	0.891 (0.964; 0.569 to 1.633)
Preoperative				
Hemoglobin, mean (SD), g/L	131.83 (19.15)	133.02 (17.12)	0.409	
Albumin, mean (SD), g/L	37.13 (4.70)	38.38 (4.61)	0.001	0.154 (0.968; 0.926 to 1.012)
NLR, median (quartiles)	2.49 (1.73, 3.58)	2.26 (1.64, 3.11)	#0.032	0.510 (1.047; 0.914 to 1.200)
PLR, median (quartiles)	146.68 (117.36, 206.66)	145.08 (111.37, 189.81)	#0.211	0.785 (0.999; 0.996 to 1.003)
Postoperative				
Hemoglobin, median (quartiles), g/L	108.00 (97.00, 121.00)	113.00 (101.00, 124.00)	#0.004	
Albumin, median (quartiles), g/L	29.75 (26.63, 32.83)	30.80 (27.73, 34.08)	#0.002	0.366 (0.979; 0.935 to 1.025)
NLR, median (quartiles)	15.96 (10.20, 23.10)	15.03 (9.95, 22.35)	#0.348	0.182 (1.015; 0.993 to 1.038)
PLR, median (quartiles)	251.50 (173.36, 379.64)	251.70 (172.92, 360.19)	#0.438	0.670 (1.000; 0.998 to 1.001)
Blood Loss, median (quartiles), ml	300.00 (200.00, 400.00)	300.00 (200.00, 400.00)	#0.011	
Duration of Surgery, median (quartiles), min	390.00 (300.00, 470.00)	360.00 (280.00, 435.00)	#0.002	0.372 (1.001; 0.999 to 1.004)
Intraoperative Fluid Infusion, median (quartiles), ml	3000 (2500, 3500)	3000 (2500, 3500)	#0.054	
Intraoperative Fluid Infusion Rate, median (quartiles), ml/(kg×h)	8.09 (6.48, 9.79)	8.17 (6.42, 9.74)	#0.829	0.360 (1.053; 0.943 to 1.176)
Intraoperative RBC Transfusion, median (quartiles), u	0.0 (0.0, 2.0)	0.0 (0.0, 2.0)	#0.005	0.042 (1.141; 1.005 to 1.295)
Intraoperative FFP Transfusion, median (quartiles), ml	0.0 (0.0, 200.0)	0.0 (0.0, 0.0)	#0.008	
Intraoperative Urine Output, median (quartiles), ml	750.0 (500.0, 1000.0)	700.0 (400.0, 1000.0)	#0.062	0.277 (1.000; 1.000 to 1.001)
Fluid Infusion over 24 hr, median (quartiles), ml	4270 (3500, 5030)	4150 (3500, 4800)	#0.173	
Fluid Infusion Rate over 24 hr, median (quartiles), ml/(kg×h)	2.97 (2.44, 3.68)	2.83 (2.35, 3.45)	#0.042	0.888 (1.022; 0.754 to 1.386)
Postoperative ICU Admission, No. (%)	33 (9.7)	9 (3.0)	0.001	0.078 (0.445; 0.181 to 1.093)
Reoperation, No. (%)	35 (10.3)	4 (1.3)	<0.001	<0.001 (0.110; 0.038 to 0.323)
Vasopressor Administration, No. (%)	75 (22.1)	71 (23.4)	0.695	0.540 (1.138; 0.753 to 1.720)

PLOS, prolonged length of stay; OR, Odds Ratio; 95% CI, 95% confidence interval; BMI, Body Mass Index; ASA, American Society of Anesthesiologists; RBC, red blood cell; FFP, free-frozen plasma; NLR, neutrophil-to-lymphocyte ratio; PLR, platelet-to-lymphocyte ratio; LMR, lymphocyte- to-monocyte ratio.

Variables in the multivariable analysis were selected by collinearity diagnostics.

# indicates that the Mann-Whitney U test was utilized.

### 3.3 Quantification of intraoperative circulation data

In order to quantify the intraoperative circulation (systolic, diastolic blood pressure, pulse and heart rate) data, a total of 114 features was extracted using machine learning-based technologies (Fourier transform, Ricker wavelet, Lempel-Ziv compression algorithm, approximation entropy, permutation entropy, linear regression following blocks aggregation, and percentage of duplicate and non-duplicate values) with 0.01 dual test fade discover rate (**Supplementary Table S1**).

### 3.4 Quantified intraoperative circulation data predicted the occurrence of PLOS

To evaluate predictive capabilities of quantified intraoperative circulation data, random forest and xgboost methods were utilized for predictive model creation due to their strengths in handling extensive feature amounts. Additionally, a comparison was made between features extracted from intraoperative circulation time series data using machine learning-based algorithms and features derived from manual intraoperative circulation evaluations. Both sets of features were utilized in building predictive models with the same algorithms, along with other relevant perioperative clinical factors.

**Figure 2:**
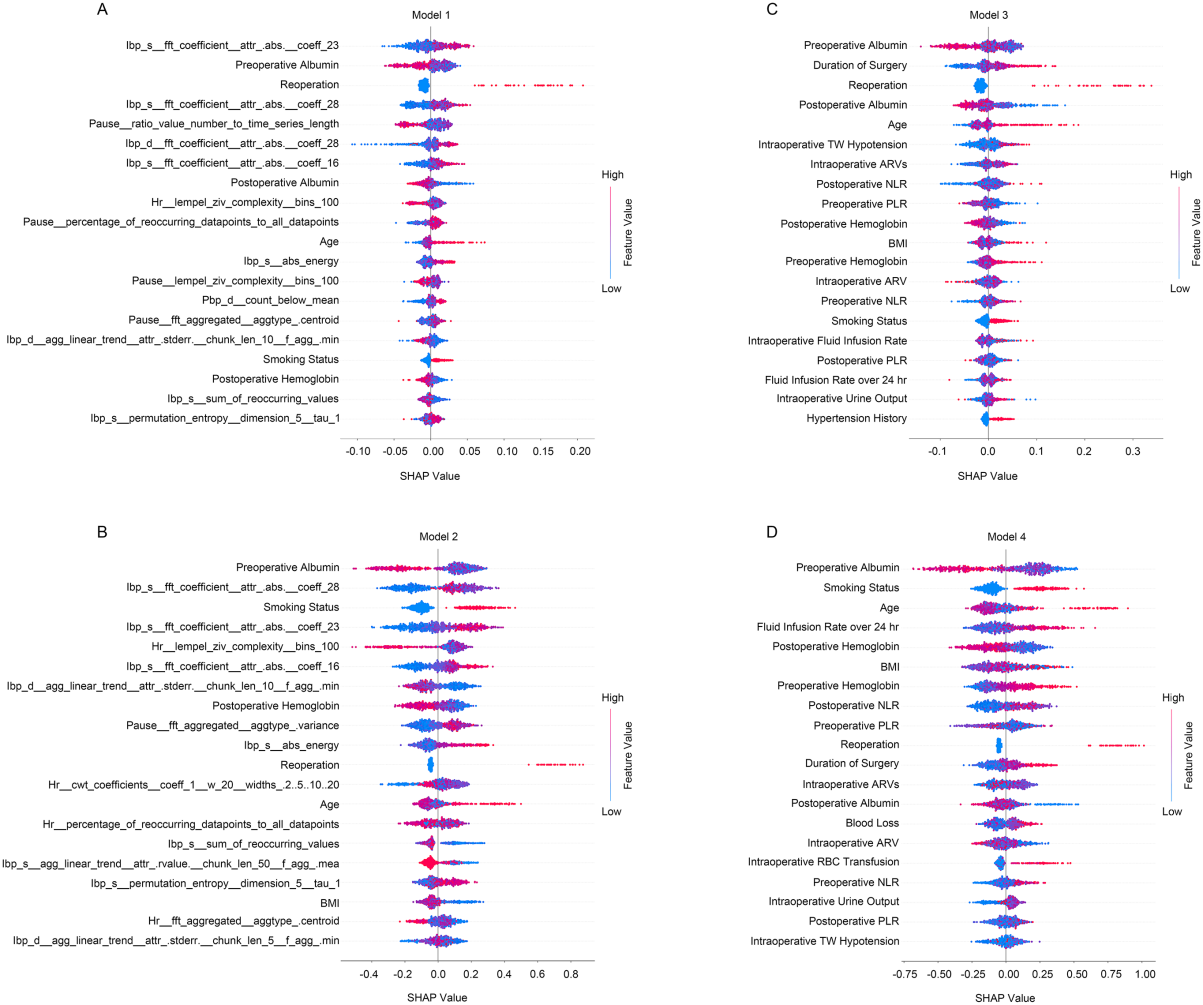
The summary of SHAP values of the top 20 important features for predictive models. **(A)** The random forest predictive model incorporating quantified intraoperative circulation data; **(B)** The xgboost predictive model incorporating quantified intraoperative circulation data; **(C)** The random forest predictive model incorporating manual intraoperative circulation assessment features; **(D)** The xgboost predictive model incorporating manual intraoperative circulation assessment features. Red indicates higher feature values and blue indicates lower feature values.

The predictive models were successfully developed, and the significance of the incorporated features was measured using SHAP values (**Figure 2**). Besides, the trend of the SHAP value of the top 10 important features in each model were shown in **Supplementary Figure S1**. In both random forest (Model 1) and xgboost (Model 2) models, specific features derived from quantified intraoperative circulation data were found to be associated with the occurrence of PLOS following free flap reconstruction (**Figures 2A, B**; **Supplementary Figures S1A, B**). On the other hand, features obtained from manual intraoperative circulation assessments such as intraoperative TW hypotension, AVR and AVRs emerged as predictive factors for PLOS (**Figures 2C, D**; **Supplementary Figures S1C, D**). Furthermore, age, smoking status, preoperative albumin level, postoperative hemoglobin level and postoperative reoperation demonstrated consistent predictive effects across all four models (**Figure 2**).

**Figure 3:**
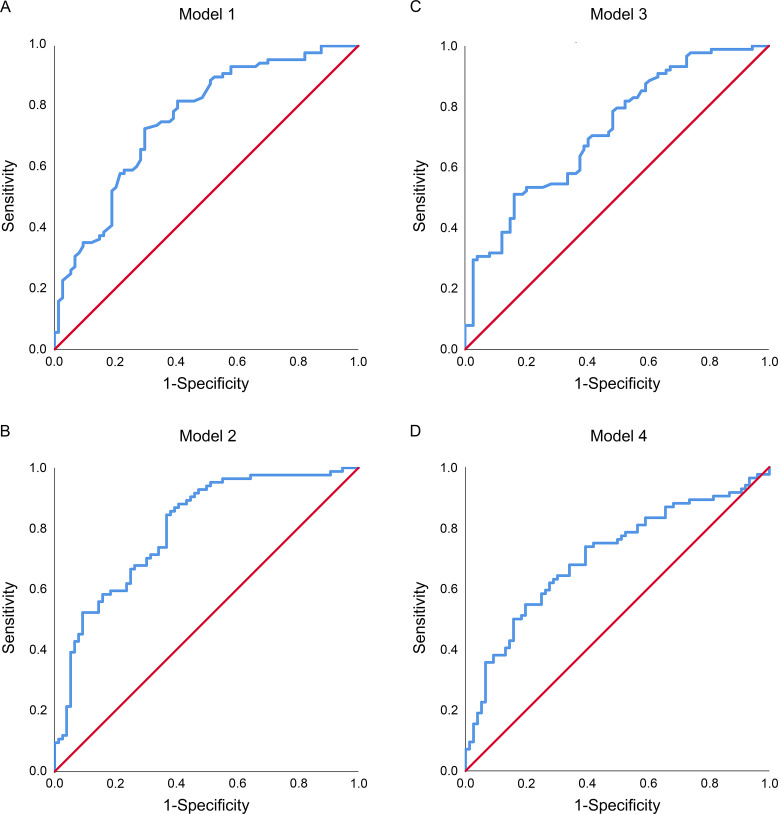
The receiver operating characteristic (ROC) curves of predictive models in the validation cohorts. **(A)** The random forest predictive model incorporating quantified intraoperative circulation data; **(B)** The xgboost predictive model incorporating quantified intraoperative circulation data; **(C)** The random forest predictive model incorporating manual intraoperative circulation assessment features; **(D)** The xgboost predictive model incorporating manual intraoperative circulation assessment features.

In our study, the accuracy of the predictive models was evaluated using ROC curves (**Figure 3**) and confusion matrix (**Supplementary Figure S2**). Comparisons were made between the random forest and xgboost models developed using both quantified intraoperative circulation data and manual intraoperative circulation assessments. The results indicated that the quantified data prediction models (the AUROC for Model 1 and Model 2 were 0.756 [95% CI, 0.682–0.831] and 0.801 [95% CI, 0.733–0.869], and the OA were 71.25% and 68.75% respectively) (**Figures 3A, B**; **Supplementary Figures S2A, B**) exhibited higher AUROC values and OA compared to the prediction models based on manual assessments (the AUROC for Model 3 and Model 4 were 0.719 [95% CI, 0.641–0.797] and 0.705 [95% CI, 0.624–0.786], and the OA were 63.13% and 65.00% respectively) (**Figures 3C, D**; **Supplementary Figures S2C, D**).

### 3.5 Real-time manner of quantified intraoperative circulation data predictive model

**Figure 4:**
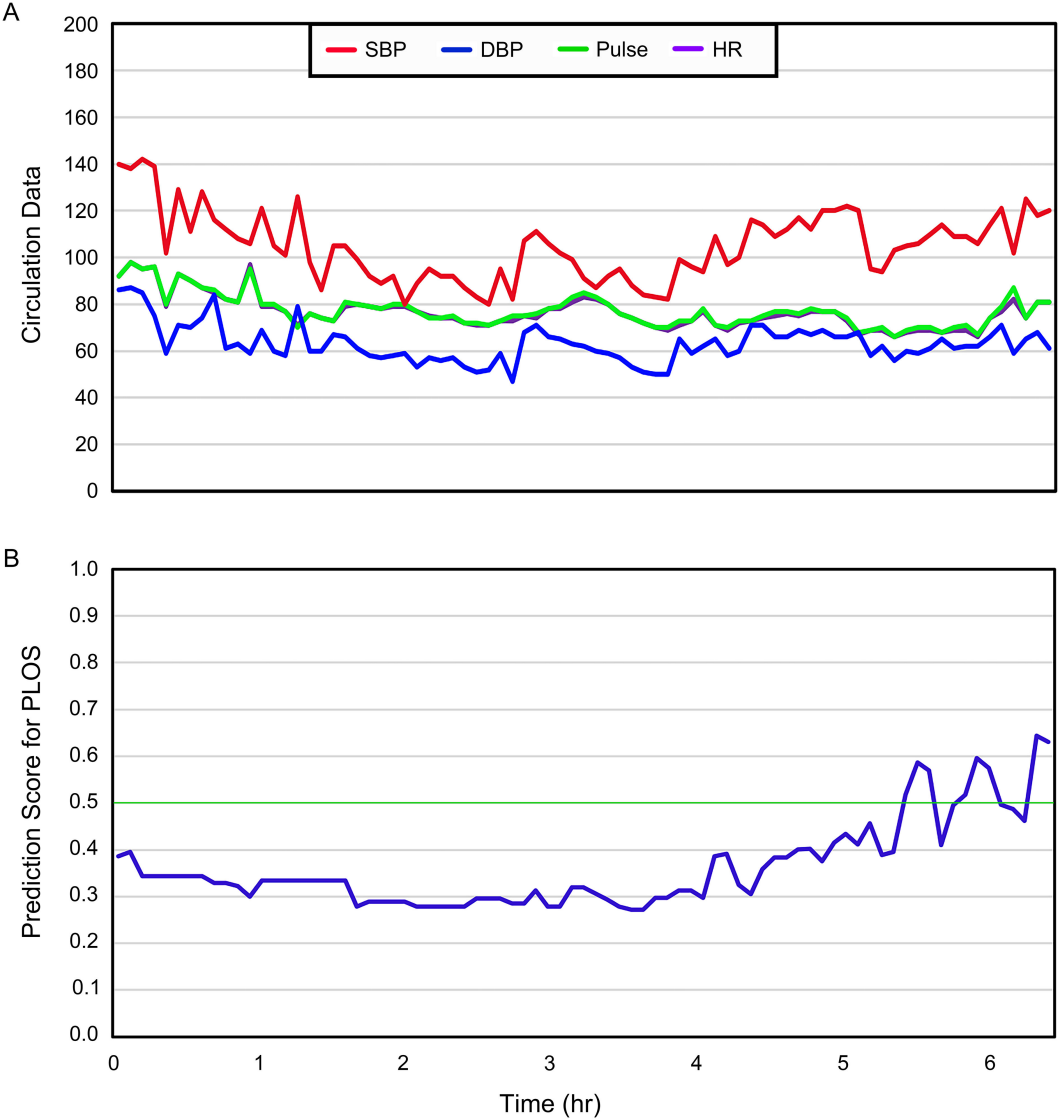
The performance of the real-time prediction system that based on the prediction score for the quantified intraoperative circulation data xgboost prediction model of a randomly selected patient. **(A)** The recorded intraoperative circulation data of the patient, including pulse, heart rate (HR), systolic blood pressure (SBP) and diastolic blood pressure (DBP); **(B)** The real-time prediction scores derived from intraoperative circulation data through machine learning algorithms.

Apart from their higher accuracy, the quantified intraoperative circulation data models also demonstrated a real-time prediction capability. **Figure 4** showcases the performance of a real-time prediction system based on the prediction score using the xgboost predictive model with quantified intraoperative circulation data. At intervals of every 5 minutes, the circulation data including heart rate, pulse, systolic and diastolic blood pressure were recorded for randomly selected patients (**Figure 4A**). With the accumulation of intraoperative circulation data, the features of intraoperative circulation data extracted using machine learning algorithms changed dynamically. Therefore, subsequently, these data inputs were used to generate prediction probability scores for postoperative outcomes in real time (**Figure 4B**). This dynamic approach allows for timely monitoring and assessment of patient risk throughout their surgical process.

## 4 Discussion

In this study, we utilized machine learning algorithms (Fourier transform, ricker wavelet, etc.) to analyze intraoperative circulation time series data and extract relevant features. Based on quantified intraoperative circulation data and other perioperative clinical factors, our research developed and validated real-time predictive models for PLOS after head and neck surgery with free flap reconstruction. The implementation of these models not only enables clinicians to identify high-risk patients susceptible to PLOS but also offers a potential method for evaluating and optimizing management strategies during free flap reconstruction.

Previous research has focused on understanding the relationship between intraoperative circulation state and postoperative prognosis (9, 15, 24). However, most studies have primarily focused on a single circulation indicator, such as intraoperative blood pressure, while overlooking the complexity of irregular time series data associated with intraoperative circulation. The high variability in these datasets poses challenges in identifying relevant features through manual methods. Advancements in machine learning and neural network algorithms offer new opportunities for feature extraction and analysis of time series data (23, 25). Therefore, our study addresses this challenge by utilizing machine learning algorithms to extract features from intraoperative circulation and assess their impact on PLOS after free flap reconstruction. Among numerous algorithms employed for intraoperative circulation data quantification, Fourier transform and wavelet analysis captured global and localized frequency patterns, while complexity and entropy measures provided insights into the structural and dynamic properties of the data. The combination of these algorithms enables a comprehensive analysis of intraoperative circulatory data.

While machine learning algorithms have proven effective in extracting features (114 features) from intraoperative circulation data, challenges persist in interpreting these features and optimizing intraoperative circulation management strategies during head and neck surgery with free flap reconstruction (23). To address this issue, the current study developed individualized predictive models using quantified intraoperative circulation data and other relevant perioperative clinical factors through machine learning algorithms (random forest and xgboost). Along with the accumulation of intraoperative circulation data, the features of quantified intraoperative circulation data varied accordingly. Dynamic changes in patients’ indices of quantified intraoperative circulation data provided the potential for predictive models that comprise these indices to dynamically assess the impact of intraoperative circulation on postoperative PLOS. Therefore, real-time prediction scores offered by these models during free flap reconstruction may serve as valuable references for clinicians to refine their intraoperative circulation management and potentially mitigate the occurrence of PLOS after free flap reconstruction.

To evaluate the predictive capabilities of two different intraoperative circulation data evaluation approaches, we developed predictive models by combining features from manual intraoperative circulation assessments with other relevant perioperative clinical factors using machine learning algorithms (random forest and xgboost). The prediction models based on quantified intraoperative circulation data demonstrated superior performance in terms of AUROC and OA when compared to the models solely relying on manual intraoperative circulation assessments. Our findings suggest that features extracted and selected from intraoperative circulation data through machine learning algorithms not only enable real-time predictions for PLOS following head and neck surgery with free flap reconstruction, but also offer a more comprehensive approach to intraoperative circulation management assessment.

Incorporating manual intraoperative circulation assessments into the predictive models showed intraoperative TW hypotension, AVR and AVRs as independent predictors of PLOS following free flap reconstruction, aligning with previous research emphasizing the impact of intraoperative hypotension on complications and the association of intraoperative MAP variability with adverse events (9, 14, 26). Although their predictive effect was relatively lower compared to quantified intraoperative circulation data, our results underscore the importance of minimizing severe intraoperative hypotension and MAP variability during head and neck surgery with free flap reconstruction.

Independent risk factors (smoking status, intraoperative RBC transfusion and postoperative reoperation) were determined for PLOS following free flap reconstruction. And variables like age, smoking status, preoperative albumin level, postoperative hemoglobin level and postoperative reoperation exhibited predictive effects on PLOS during modeling. Demographic characteristics (age and smoking status) and postoperative reoperation impacted postoperative short-term prognoses (27, 28). Besides, our prior work established robust links between intraoperative RBC transfusion, perioperative nutrient level and the occurrence of complications and PLOS following fibular flap reconstructions (29–31). Integrating these variables boosted the models’ predictive strength, underscoring their essential role in precise PLOS prediction.

Several limitations should be acknowledged in our study. Firstly, as with retrospective analyses, the possibility of unaccounted confounders exists. Secondly, the 5-minute interval of collected intraoperative circulation data might not capture important fluctuations, impacting our analysis. Thirdly, dividing patients into primary and validation cohorts to create predictive models led to reduced sample size. Lastly, while numerous machine learning and deep learning algorithms exist for time series analysis, their application to predict intraoperative circulation data’s influence on PLOS warrants further exploration. In light of these limitations, future research endeavors should focus on prospective studies with larger sample sizes and employ more robust machine learning or deep learning algorithms to better forecast the likelihood of PLOS based on perioperative variables after free flap reconstruction.

This study used machine learning algorithms to extract intraoperative circulation data characteristics and create real-time personalized predictive models for PLOS following head and neck surgery with free flap reconstruction. Our results provide new insights into assessing the connection between intraoperative circulation management and adverse events and suggest possibilities for enhancing intraoperative circulation management through real-time prediction scores. Moreover, our predictive models integrated intraoperative circulation data features and clinical risk factors, ensuring precise estimation of a patient’s PLOS development likelihood.

## Data Availability

The raw data supporting the conclusions of this article will be made available by the authors, without undue reservation.
