# Supplementary material for: Intraoperative circulation predict prolonged length of stay after head and neck free flap reconstruction: a retrospective study based on machine learning
Source: Front Oncol. 2025 Jan 10;14:1473447. doi: 10.3389/fonc.2024.1473447 (PMC11757266; doi:10.3389/fonc.2024.1473447)
Supplement: Supplementary file 1 [file DataSheet1.docx]

Supplementary Material

# Supplementary Table

**Table S1.** Features Extracted from Intraoperative Circulation Data through Machine Learning-Based Technologies.

| **Features** | **Features** |
| --- | --- |
| ibp_d__lempel_ziv_complexity__bins_2 | pause__agg_linear_trend__attr_.stderr.__chunk_len_10__f_agg_.max |
| ibp_s__sum_values | ibp_s__fft_coefficient__attr_.abs.__coeff_28 |
| ibp_s__fft_coefficient__attr_.abs.__coeff_0 | pause__number_peaks__n_3 |
| ibp_s__fft_coefficient__attr_.real.__coeff_0 | hr__agg_linear_trend__attr_.stderr.__chunk_len_10__f_agg_.max |
| pause__count_below_mean | ibp_d__lempel_ziv_complexity__bins_3 |
| hr__count_below_mean | ibp_s__permutation_entropy__dimension_5__tau_1 |
| ibp_s__permutation_entropy__dimension_7__tau_1 | hr__lempel_ziv_complexity__bins_100 |
| ibp_d__count_below_mean | ibp_s__agg_linear_trend__attr_.stderr.__chunk_len_50__f_agg_.var |
| ibp_d__permutation_entropy__dimension_7__tau_1 | ibp_s__sum_of_reoccurring_values |
| ibp_d__length | ibp_s__number_cwt_peaks__n_5 |
| ibp_d__range_count__max_1000000000000.0__min_0 | ibp_d__lempel_ziv_complexity__bins_10 |
| hr__range_count__max_1000000000000.0__min_0 | hr__percentage_of_reoccurring_datapoints_to_all_datapoints |
| ibp_s__length | pause__number_peaks__n_1 |
| pause__length | ibp_d__agg_linear_trend__attr_.stderr.__chunk_len_50__f_agg_.var |
| ibp_s__range_count__max_1000000000000.0__min_0 | pause__permutation_entropy__dimension_6__tau_1 |
| pause__range_count__max_1000000000000.0__min_0 | ibp_s__agg_linear_trend__attr_.stderr.__chunk_len_50__f_agg_.max |
| hr__length | ibp_s__number_peaks__n_5 |
| hr__linear_trend__attr_.stderr | ibp_d__agg_linear_trend__attr_.stderr.__chunk_len_50__f_agg_.max |
| ibp_s__abs_energy (10^6^) | pause__lempel_ziv_complexity__bins_100 |
| pause__permutation_entropy__dimension_7__tau_1 | ibp_s__number_peaks__n_3 |
| ibp_s__fft_aggregated__aggtype_.variance | ibp_d__agg_linear_trend__attr_.stderr.__chunk_len_10__f_agg_.min |
| ibp_s__permutation_entropy__dimension_6__tau_1 | pause__number_peaks__n_5 |
| pause__agg_linear_trend__attr_.stderr.__chunk_len_5__f_agg_.max | ibp_s__absolute_sum_of_changes |
| ibp_s__fft_aggregated__aggtype_.centroid | hr__fft_coefficient__attr_.abs.__coeff_0 |
| pause__number_cwt_peaks__n_5 | hr__fft_coefficient__attr_.real.__coeff_0 |
| ibp_d__fft_aggregated__aggtype_.variance | hr__sum_values |
| ibp_s__number_cwt_peaks__n_1 | pause__fft_coefficient__attr_.real.__coeff_0 |
| pause__fft_aggregated__aggtype_.variance | pause__fft_coefficient__attr_.abs.__coeff_0 |
| hr__agg_linear_trend__attr_.stderr.__chunk_len_5__f_agg_.max | pause__sum_values |
| pause__lempel_ziv_complexity__bins_2 | pause__agg_linear_trend__attr_.stderr.__chunk_len_10__f_agg_.mea |
| pause__ratio_value_number_to_time_series_length | pause__percentage_of_reoccurring_values_to_all_values |
| pause__percentage_of_reoccurring_datapoints_to_all_datapoints | pause__fft_aggregated__aggtype_.centroid |
| ibp_s__count_below_mean | hr__agg_linear_trend__attr_.stderr.__chunk_len_10__f_agg_.min |
| ibp_d__fft_aggregated__aggtype_.centroid | ibp_d__count_above_mean |
| ibp_s__lempel_ziv_complexity__bins_2 | pause__number_peaks__n_10 |
| hr__agg_linear_trend__attr_.stderr.__chunk_len_5__f_agg_.min | hr__agg_linear_trend__attr_.stderr.__chunk_len_10__f_agg_.mean |
| pause__linear_trend__attr_.stderr | ibp_s__fft_coefficient__attr_.abs.__coeff_16 |
| ibp_d__permutation_entropy__dimension_6__tau_1 | ibp_d__linear_trend__attr_.stderr |
| ibp_d__number_peaks__n_1 | ibp_s__agg_linear_trend__attr_.stderr.__chunk_len_50__f_agg_.mea |
| hr__agg_linear_trend__attr_.stderr.__chunk_len_5__f_agg_.mean | ibp_d__agg_linear_trend__attr_.stderr.__chunk_len_50__f_agg_.mea |
| ibp_s__number_peaks__n_1 | ibp_d__lempel_ziv_complexity__bins_5 |
| hr__lempel_ziv_complexity__bins_2 | ibp_d__approximate_entropy__m_2__r_0.3 |
| pause__agg_linear_trend__attr_.stderr.__chunk_len_5__f_agg_.mean | ibp_d__fft_coefficient__attr_.abs.__coeff_28 |
| ibp_s__sum_of_reoccurring_data_points | ibp_s__lempel_ziv_complexity__bins_10 |
| hr__ratio_value_number_to_time_series_length | ibp_s__agg_linear_trend__attr_.rvalue.__chunk_len_50__f_agg_.mea |
| ibp_d__fft_coefficient__attr_.real.__coeff_0 | hr__agg_linear_trend__attr_.stderr.__chunk_len_50__f_agg_.mean |
| ibp_d__fft_coefficient__attr_.abs.__coeff_0 | ibp_d__number_peaks__n_5 |
| ibp_d__sum_values | hr__cwt_coefficients__coeff_0__w_20__widths_.2..5..10..20 |
| hr__fft_aggregated__aggtype_.variance | hr__fft_aggregated__aggtype_.centroid |
| pause__sum_of_reoccurring_data_points | ibp_d__absolute_sum_of_changes |
| ibp_d__number_peaks__n_3 | ibp_d__agg_linear_trend__attr_.stderr.__chunk_len_5__f_agg_.min |
| pause__agg_linear_trend__attr_.stderr.__chunk_len_5__f_agg_.min | pause__agg_linear_trend__attr_.stderr.__chunk_len_10__f_agg_.min |
| ibp_s__count_above_mean | pause__number_cwt_peaks__n_1 |
| hr__sum_of_reoccurring_data_points | pause__cwt_coefficients__coeff_0__w_20__widths_.2..5..10..20 |
| ibp_d__sum_of_reoccurring_data_points | hr__cwt_coefficients__coeff_1__w_20__widths_.2..5..10..20 |
| hr__permutation_entropy__dimension_7__tau_1 | ibp_d__permutation_entropy__dimension_5__tau_1 |
| ibp_s__fft_coefficient__attr_.abs.__coeff_23 | pause__agg_linear_trend__attr_.stderr.__chunk_len_50__f_agg_.mea |

## Supplementary Figures


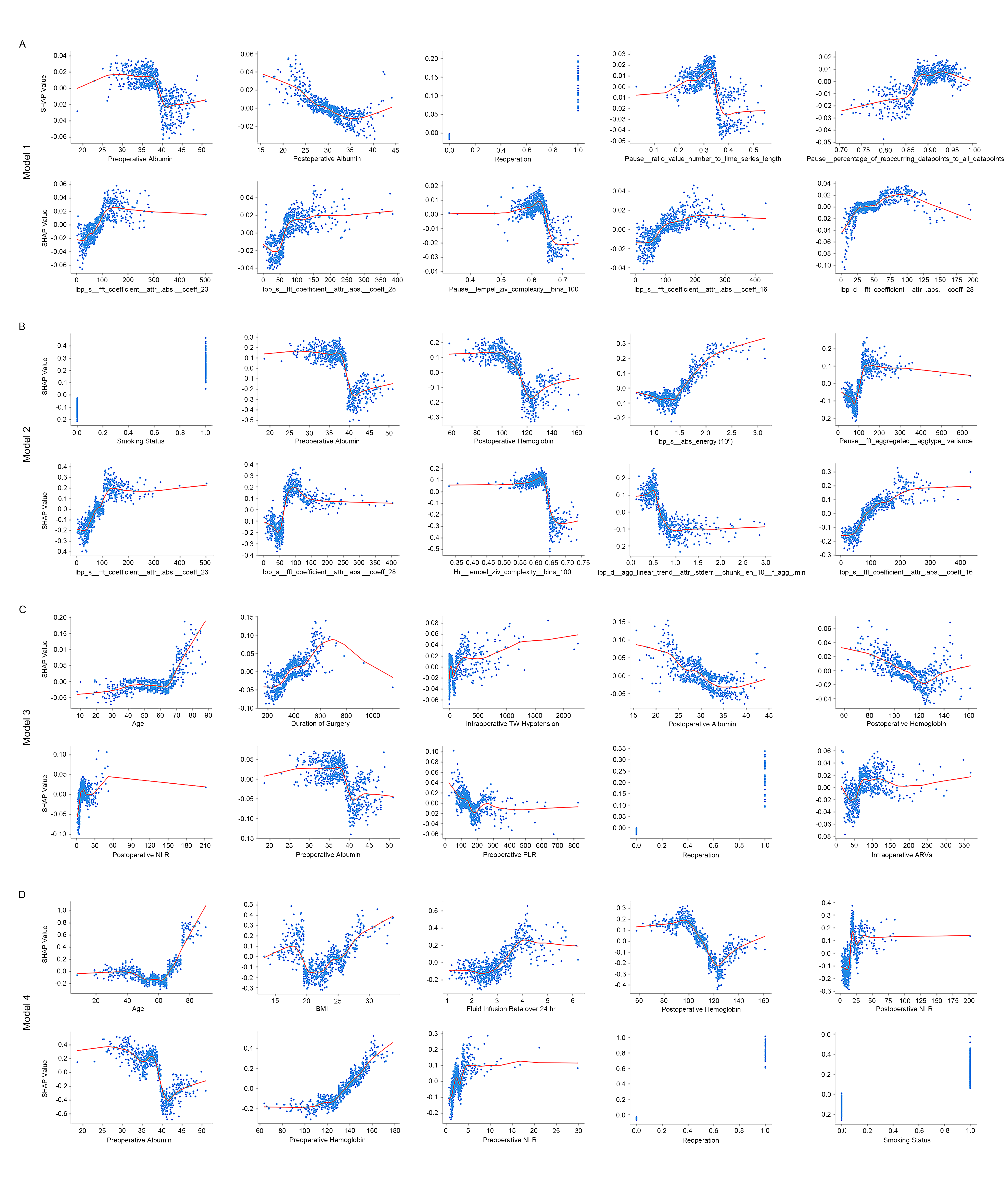


**Figure S1.** The trend of the SHAP value of the top 10 important features for postoperative PLOS predictive models. (A) The random forest predictive model incorporating quantified intraoperative circulation data; (B) The xgboost predictive model incorporating quantified intraoperative circulation data; (C) The random forest predictive model incorporating manual intraoperative circulation assessment features; (D) The xgboost predictive model incorporating manual intraoperative circulation assessment features.


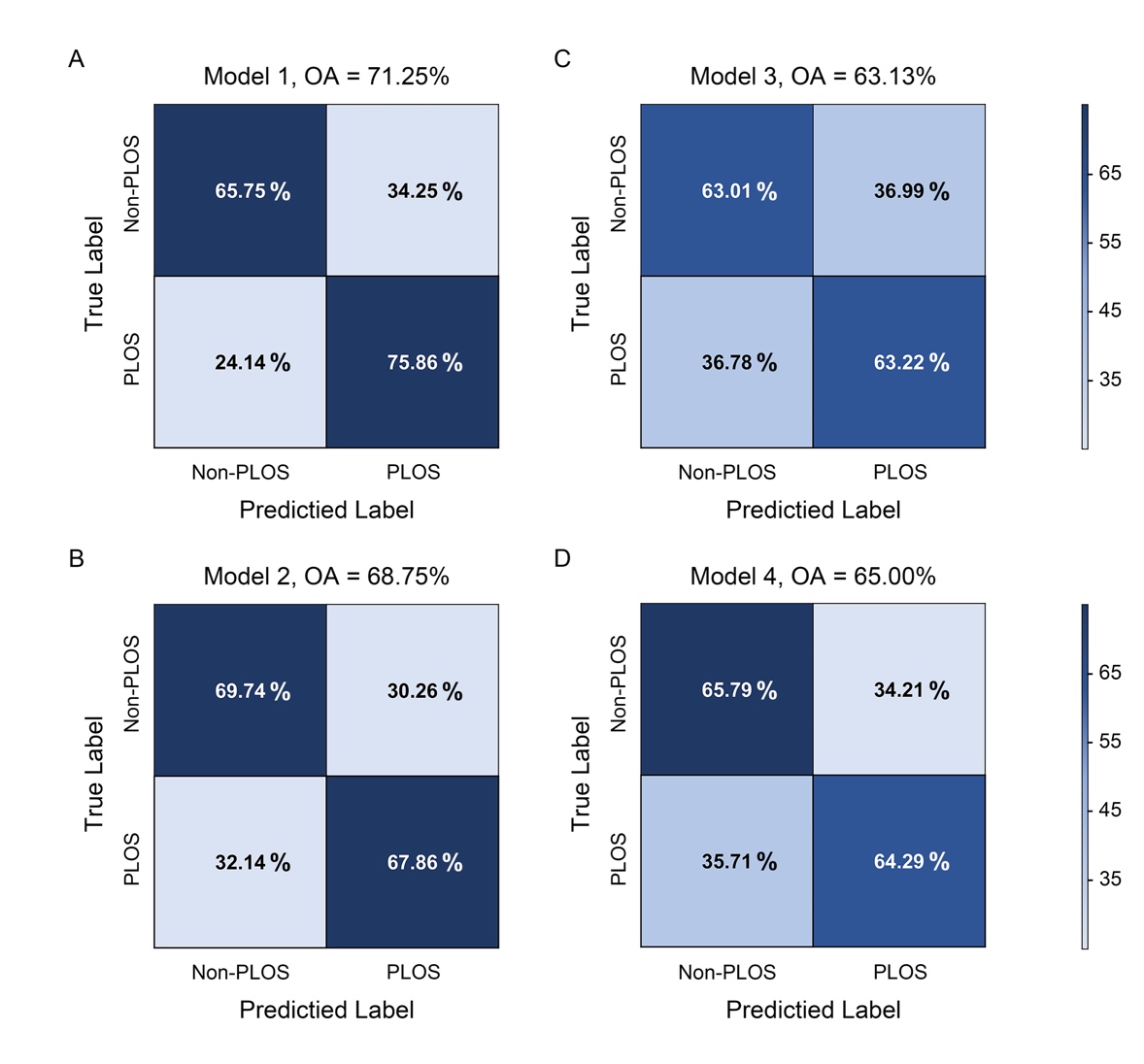


**Figure S2.** The confusion matrix and overall accuracy (OA) of predictive models in the validation cohorts. (A) The random forest predictive model incorporating quantified intraoperative circulation data; (B) The xgboost predictive model incorporating quantified intraoperative circulation data; (C) The random forest predictive model incorporating manual intraoperative circulation assessment features; (D) The xgboost predictive model incorporating manual intraoperative circulation assessment features.
